# Combination of High Hydrostatic Pressure and Ultrafiltration to Generate a New Emulsifying Ingredient from Egg Yolk

**DOI:** 10.3390/molecules25051184

**Published:** 2020-03-05

**Authors:** Mélanie Giarratano, Pauline Duffuler, Julien Chamberland, Guillaume Brisson, James D. House, Yves Pouliot, Alain Doyen

**Affiliations:** 1 Department of Food Sciences, Institute of Nutrition and Functional Foods (INAF), Université Laval, Quebec, QC G1V 0A6, Canada; Melanie.Giarratano.1@ulaval.ca (M.G.); Guillaume.Brisson@fsaa.ulaval.ca (G.B.); Yves.Pouliot@fsaa.ulaval.ca (Y.P.); 2 Department of Agricultural, Food and Nutritional Science, University of Alberta, Edmonton, AB T6G 2P5, Canada; duffuler@ualberta.ca; 3 Department of Food and Human Nutritional Sciences, University of Manitoba, Winnipeg, MB R3T 2N2, Canada; James.House@umanitoba.ca

**Keywords:** phosvitin, high hydrostatic pressure, granule, ultrafiltration, egg yolk, emulsifying properties

## Abstract

Egg yolk granule phosvitin (45 kDa) is a phosphoprotein known for its emulsifying properties. Recently, high hydrostatic pressure (HHP) treatment of granule induced the transfer of phosvitin to the soluble plasma fraction. This project evaluated the performance of the ultrafiltration (UF) used to concentrate phosvitin from the plasma fraction to produce a natural emulsifier. Phosvitin was characterized in plasma from a pressure-treated granule (1.73 ± 0.07% *w*/*w*) and in its UF retentate (26.00 ± 4.12% *w*/*w*). The emulsifying properties of both retentates were evaluated. The emulsion prepared with phosvitin-enriched retentate was more resistant to flocculation and creaming. Confocal laser scanning microscopy showed a network of aggregated protein similar to a gel, which encapsulated oil droplets in emulsions made with UF-retentate of plasma from pressure-treated granule. However, although sodium dodecyl sulfate-polyacrylamide gel electrophoresis (SDS-PAGE) showed that β-phosvitin is recovered in the cream, it is difficult to attribute the improved emulsifying properties of the UF-retentate of plasma from pressure-treated granules only to phosvitin.

## 1. Introduction

Phosvitin represents 4% *w*/*w* of the dry matter and 11% *w*/*w* of the protein in egg yolk [1]. Present in the granule fraction as non-soluble high-density lipoprotein (HDL)-phosvitin complexes linked by phosphocalcic bonds, phosvitin is generated by the cleavage of vitellogenin [2] and is found in three isoforms: α1, α2 and β-phosvitin [3,4]. These isoforms are composed of three to four subunits of 35 to 40 kDa for α-phosvitins, and four to five subunits of 45 kDa for β-phosvitin [3]. More than half of phosvitin amino acids are serines (125 serine residues of a total of 217 amino acids), and 90% of the serines are phosphorylated [5]. The high phosphoserine concentration in the center of the protein gives phosvitin a very hydrophilic center, a strongly negative charge (−179 mV at neutral pH) and a linear structure, due to electrostatic repulsions that prevent folding [6,7].

Due to its negative charge, phosvitin has a high binding capacity for iron [8,9]. As a consequence, phosvitin and the phosphopeptides generated from its enzymatic hydrolysis exhibit high antioxidant activities [4,10,11,12]. Phosvitin is also recognized for its antimicrobial activity [13], its action against melanogenesis [14,15,16], and its ability to improve the bioavailability of calcium in the intestine and the absorption of calcium by bones [17,18,19,20]. Some studies have also investigated the emulsifying properties of phosvitin [10,21,22,23,24,25]. Phosvitin’s hydrophobic terminal structures can adsorb at the interface of oil droplets to stabilize emulsions [10]. Several parameters such as pH, ionic strength, degree of aggregation, freeze/thaw cycles or heat treatment influence the emulsifying properties of phosvitin [21,22,23,25,26,27]. However, phosvitin does not express its emulsifying character in egg yolk. Instead, low-density lipoprotein (LDL), which has stronger interactions with hydrophobic proteins, is found at the interface of oil droplets and stabilizes emulsions [24,28].

Due to its biological and functional properties, several studies have focused on phosvitin extraction techniques. Usually, phosvitin extraction from the granule is carried out using sodium chloride (NaCl) or ammonium sulphate ((NH_4_)_2_SO_4_) which breaks the phosphocalcic bridges of the HDL-phosvitin complex and releases the phosphoprotein from the granular matrix. The fractions obtained are further purified using ethanol [29], heat treatment [4,30] or anion exchange chromatography [10,31,32]. Anion exchange chromatography is particularly interesting for the purification of phosvitin, with purification rates ≥92% [10,31,32]. Castellani et al. [10] also recovered 85% of phosvitin from the granule using 0.17 M NaCl, 0.9M MgSO_4_ and several centrifugation steps. They purified the fraction by up to 98% using anion exchange chromatography [10]. Unfortunately, this technique uses organic solvents and may be time-consuming and costly, despite the excellent extraction yields and purification rates. Accordingly, this technique may not be well adapted to the food industry and is inconsistent with current strategies on environmental protection and sustainable development. For this reason, a cleaner technique is needed for the extraction and the purification of phosvitin.

High hydrostatic pressure (HHP) is an ecofriendly technology that has been used in the food industry since the 1990s to reduce the microbial load in various food products [33]. Since HHP does not involve heat intervention, the nutritional and organoleptic properties of treated foods are preserved [33,34,35,36]. Recently, Naderi et al. [37,38] proposed the use of HHP as a pre-treatment for egg yolk and granule to improve the extraction of folic acid (5-MTHF). HHP caused disintegration of the granular network and changed the composition of each fraction; 5-MTHF and phosvitin initially present in granule were both released from the network and found in the plasma [37,38]. Egg yolk granule has a very compact and poorly hydrated structure, mainly due to the non-soluble HDL-phosvitin complex [39,40,41,42]. It was hypothesized that the application of pressure to granule induces the entry of water into the network, thus solubilizing the phosphocalcic bridges, and allowing the transfer of phosvitin into the soluble plasma. Treatment of the granule fraction at 400 MPa for 5 min allowed the most efficient extraction of 5-MTHF, with a recovery of 78% in plasma. However, the protein profile produced by sodium dodecyl sulphate–polyacrylamide gel electrophoresis (SDS–PAGE) showed that the phosvitin band intensity was higher for the 600 MPa, 10 min treatment of the granule fraction [38]. Duffuler [43] demonstrated that plasma obtained by pressurizing the granule fraction had the highest concentration (33.3 ± 4.39% *w*/*w* in the dry matter) and purity (40.1 ± 3.50%) of phosvitin using the same pressure treatment [43]. Additionally, no aggregation or insolubility of phosvitin were observed using these conditions [43]. Furthermore, Castellani et al. [44] revealed the resistance of phosvitin to denaturation under high pressure, since it could still strongly bind iron after a high-pressure treatment (300 to 600 MPa for 10 min). Nevertheless, the plasma containing phosvitin generated by this method was quite dilute, requiring concentration to obtain an enriched fraction. Membrane filtration is an efficient technology for the selective separation of molecules and the concentration of proteins that could be used to concentrate the plasma fraction. It has the advantage of being less energy-demanding than other concentration methods such as drying or evaporation, which require heat to remove water.

To the best of our knowledge, only Chay Pak Ting et al. [45] investigated the use of polyethersulfone (PES) ultrafiltration (UF) membrane (molecular weight cut-off [MWCO] of 10 and 30 kDa) for the concentration and purification of phosvitin. Fractionation by UF (50 °C, volume concentration factor (VCF) of 6×) and diafiltration (DF) (10 diavolumes, VCF of 11×) produced a phosvitin recovery rate of 84% in the retentate. The ratio of nitrogen (N) to phosphorus (P) of the starting phosvitin solution was 31.13, while retentates gave ratios of 7.58 and 6.64 with the 10 kDa and 30 kDa membranes, respectively, significantly improving phosvitin’s purity.

Therefore, it is hypothesized that treatment of the egg yolk granule by HHP, followed by UF concentration of the plasma fraction, would generate an ingredient with improved emulsifying properties due to high phosvitin content. The composition of the enriched plasma fraction was evaluated and validated by proteomic analysis, and its emulsifying properties were determined and compared to a non-pressure-treated plasma fraction.

## 2. Materials and Methods

### 2.1. Granule Preparation

Fresh large-sized eggs were purchased in a local grocer’s shop and stored at 4 °C until use. Egg yolk was prepared according to the methodology described by Naderi et al. [46] The egg white fraction was manually separated from the yolk. The residues of white were eliminated using filter paper. The membrane vitelline was removed with tweezers to recover only yolk. The yolk was then diluted at a 1:1 ratio with distilled water and was separated by centrifugation at 10,000× *g* for 45 min at 4 °C. The plasma fraction was removed, and the granule fraction was freeze-dried and stored at −30 °C until used.

### 2.2. High Hydrostatic Pressure Treatment

The granule was resolubilized in distilled water (10% *w*/*w*) overnight at 4 °C with constant stirring. The granule solutions were placed in hermetically sealed polyamide/polyethylene bags. A pressurization treatment (Hiperbaric 135, Hiperbaric, Burgos, Spain) at 400 MPa for 5 min was selected for this experiment. Although Naderi et al. [38] and Duffuler [43] showed that a treatment at 600 MPa for 10 min led to better phosvitin extraction, preliminary tests detected the formation of protein aggregates at a pressure of 600 MPa, which would have negatively affected UF performance [38,43]. Moreover, Duffuler [43] demonstrated that there was no significant difference in phosvitin concentration or purity between plasmas obtained by granule pressure-treated at 400 MPa for 5 min or 10 min [43].

**Figure 1:**
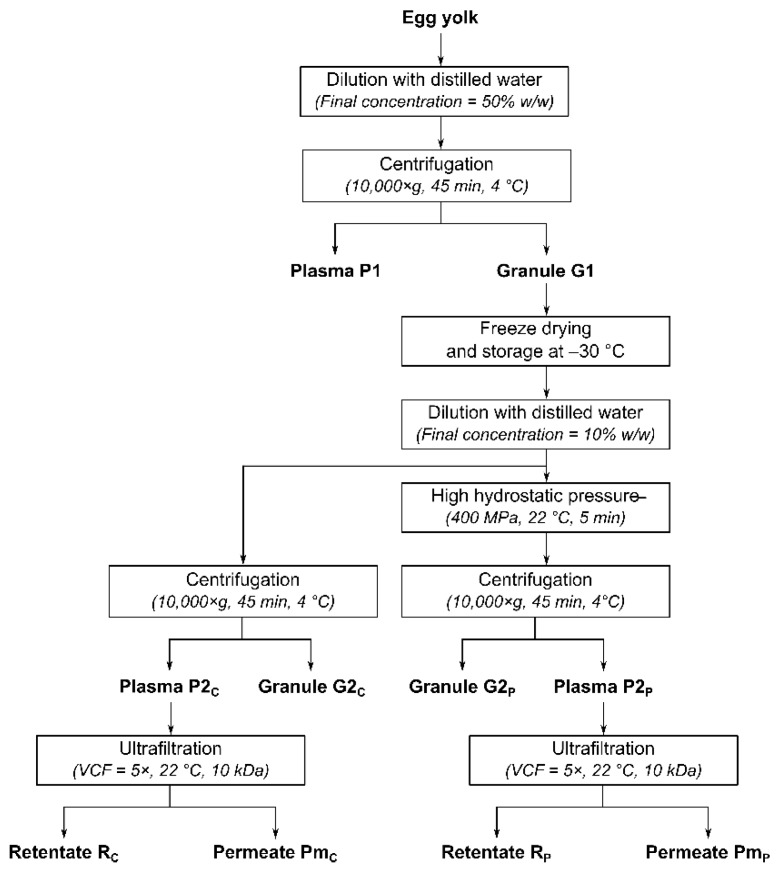
Experimental design for production of the different fractions resulting from high hydrostatic pressure (HHP) treatment of egg yolk granule, and concentration of the resulting plasma by ultrafiltration (UF). P1: initial plasma, G1: initial granule, G2_C_: control granule, P2_C_: control plasma, G2_P_: pressure-treated granule, P2_P_: plasma from pressure-treated granule, Pm_C_: control UF-permeate, R_C_: control UF-retentate, Pm_P_: UF-permeate of plasma from pressure-treated granule, R_P_: UF-retentate of plasma from pressure-treated granule.

The pressure-treated granule samples were separated by centrifugation at 10,000× *g* for 45 min, to obtain a granule (G2_P_) and a plasma (P2_P_). Non-pressurized controls of plasma (P2_C_) and granule (G2_C_) were generated using the same experimental design (Figure 1).

### 2.3. Ultrafiltration

UF experiments were adapted from Chay Pak Ting et al. [45] A laboratory scale tangential flow filtration (TFF) system (Millipore Sigma, Oakville, ON, Canada) was used with a PES membrane having a molecular weight cut-off (MWCO) of 10 kDa and a membrane surface of 0.005 m^2^ (Biomax^®^ membrane, Millipore Sigma, Oakville, ON, Canada). Following the first membrane cleaning, the pure water flux was of 288 L/m^2^·h. Optimal transmembrane pressure was determined at 96 kPa, at room temperature, for both control and plasma from pressure-treated granule P2. A volume of 400 mL of plasma samples was concentrated by UF until the maximum VCF was obtained, at room temperature. Permeate flux was measured at VCFs of 1×, 2×, 3×, 4× and 5×. The UF-retentates and UF-permeates recovered at the end of UF experiments were freeze-dried and stored at −30 °C until further use.

### 2.4. Compositional Analysis

The protein content was determined with a nitrogen gas analyzer system (LECO FP-528, Model 601-500, LECO, St. Joseph, MI, United States), based on the Dumas principle. A factor of 6.25 was used to convert the total nitrogen into protein content [46]. The lipid content was determined with the Mojonnier method (AOAC International 925.32). The mineral content was obtained by inductively coupled plasma optical emission spectrometry (ICP-OES) (Agilent 5110 ICP-OES, Agilent Technologies, Inc., Santa Clara, CA, United States). Briefly, samples were dried at 100 °C for five hours and burnt for five min on a hot plate with few drops of nitric acid (75% *v*/*v*), before being placed in a furnace at 550 °C overnight. The ashes were resolubilized in 10 mL of nitric acid (25% *v*/*v*) prior to ICP-OES.

#### 2.4.1. Quantification of Phosvitin

The phosvitin concentration in each fraction was determined through fast protein liquid chromatography (FPLC Akta Avant, GE Healthcare Bio-Sicences, Baie-D’Urfé, QC, Canada), with a HiTrap QFF anion exchange column (GE Healthcare Bio-Sciences, PA, USA). The samples were prepared as described by Duffuler [43]. Briefly, samples were solubilized at 1 mg/mL in buffer A (50 mM Tris-HCl, pH 8.0). The column was equilibrated with 2 column volumes (CV) of 100% buffer A, followed with 15 CVs of 35% buffer B (50 mM Tris-HCl, 1 M NaCl, pH 8.0) and finally with 10 CVs of 45% buffer B. Before injection, the samples were filtered through a 0.45 µm nylon membrane (Millipore Sigma, Oakville, ON, Canada). The flow rate was set at 2 mL/min and detection was performed by UV at 215 nm. A standard curve was generated with phosvitin standards (Millipore Sigma, Oakville, ON, Canada) prepared in buffer A (0.1, 0.5, 1.0 and 2.0 mg/mL), to identify and quantify phosvitin in samples.

#### 2.4.2. Protein Profiles by SDS-PAGE

Protein profiles were performed by a sodium dodecyl sulfate polyacrylamide gel electrophoresis (SDS-PAGE) analysis according to Naderi et al. [46] Freeze-dried samples were resolubilized in distilled water (1% *w*/*w*) overnight and further diluted 1:5 in distilled water. Samples were then mixed with Laemmli sample buffer containing β-mercaptoethanol (5% *v*/*v*) and heated for five min in a boiling water bath. Commercially available precast linear 4–20% gradient polyacrylamide gels (Bio-Rad Laboratories Ltd., Mississauga, ON, Canada), with a running buffer containing 10% *v*/*v* of Tris/glycine/SDS, were used for protein separation (30 mA). Gels were stained with a solution of Coomassie blue, supplemented with 0.1 M aluminum nitrate (Al(NO_3_)_3_), for the detection of phosphoproteins and destained with a solution of 10% *v*/*v* acetic acid, 40% *v*/*v* methanol and 50% *v*/*v* distilled water. Protein molecular weights were estimated with commercial molecular-weight marker (Precision plus protein^TM^, Bio-Rad Laboratories Ltd., Mississauga, ON, Canada) and protein bands were analyzed with a Chemidoc MP Imaging System (Bio-Rad Laboratories Ltd., Mississauga, ON, Canada).

#### 2.4.3. Proteomic Analysis

Some fractions (P2_P_, pressure-treated granules (R_P_) and UF-permeate of plasma from pressure-treated granule (Pm_P_)) were analyzed by liquid chromatography coupled to mass spectroscopy (LC-MS/MS) at the proteomics platform of the Research Center of the Centre hospitalier universitaire (CHU) de Québec (Quebec City, QC, Canada). Briefly, the samples were freeze-dried for transport. The products of a tryptic digestion of each sample were separated by online reversed-phase (RP) nanoscale capillary liquid chromatography (nanoLC) and analyzed by electrospray mass spectrometry (ES MS/MS). Data generated was analyzed with Mascot software (version 2.5.1, Matrix Science, London, UK). Scaffold software (version Scaffold_4.7.1, Proteome Software Inc., Portland, OR, USA) was used to validate MS/MS-based peptide and protein identifications. In Scaffold, the protein and peptide false discovery rates (FDR) were set at 1.0%, and the minimum number of peptide occurrence at 1.

### 2.5. Determination of Emulsifying Properties

#### 2.5.1. Emulsion Preparation

Oil-in-water emulsions with an oil fraction volume were made with control UF-retentate (R_C_) and UF-retentate of plasma from pressure-treated granules (R_P_) and sunflower oil. The mixture of 30 mL was first pre-mixed with an Ultra-Turrax^®^ T-25 (IKA, Werke Staufen, Germany), using a S25N-25F dispersing tool (IKA, Werke Staufen, Germany) for one min at 9500 rpm. This pre-emulsion mixture was then homogenized with a high-pressure valve homogenizer EmulsiFlex C-5 Avestin^®^ (ATA Scientific Instruments, Taren Point, Australia) at two pressure stages; first at 20 MPa, then at 5 MPa, to obtain a very fine emulsion. Each pressure stage was maintained for 5 min.

#### 2.5.2. Particle size Distribution

The volume-surface area average diameter distribution (d_[3,2]_) and the volume frequency distribution (d_[4,3]_) of the emulsion oil droplets were determined by laser light diffraction (Mastersizer 3000 Hydro, Malvern Instruments Ltd., Worcestershire, UK) at three different times after emulsion preparation (0, 4 and 24 h). In separate analyses, SDS at 1% *w*/*w* was added to evaluate the resistance of emulsions to flocculation. The addition of a drop of this denaturing agent allowed the oil droplets to de-flocculate. All of the analyses described above were done with emulsions at room temperature.

#### 2.5.3. Creaming Index

A volume of 8 mL of fresh emulsion (H_E_) was placed in a graduated tube. After 24 h at room temperature, the tube was centrifuged (3,000× *g*, 30 min, 4 °C). The volume of the dissociated phases was recorded: oil droplets in the cream at the top, oil droplets remaining in the emulsion at the center and the clear aqueous phase at the bottom (H_L_). The creaming index (%) was calculated using Equation (1):

(1) $$CI=100\times \frac{H_{L}}{H_{E}}$$

#### 2.5.4. Protein Profiles of Emulsion Fractions

The cream generated during the measurement of the creaming index was recovered using a spatula, as well as the aqueous phase below. Protein profiles of the emulsions, the creams and the aqueous phases were generated from SDS-PAGE (see Section 2.4.2).

#### 2.5.5. Confocal Laser Scanning Microscopy

A volume of 2 mL of fresh emulsion was slowly mixed with 2 mL of a 50% *w*/*v* sucrose solution. Using a syringe, the mixture was placed very gently at the bottom of a tube containing 8 mL of distilled water and centrifuged (3,000× *g*, 1 h, 4 °C). This procedure was used to wash the oil droplet from the dispersing phase. After centrifugation, the washed oil droplets in the cream fraction at the top of the tube were recovered with a spatula. This cream sample was marked with two fluorophores: 100 µL of cream was mixed with 6 µL of Fast Green in distilled water (1 mg/mL) and 2 µL of Nile Red in acetone (1 mg/mL).

After standing for 20 min, each solution was gently mixed with 200 µL of 0.5% *w*/*w* agarose. On a hot plate at 40 °C, a SecureSeal™ adhesive sheet (Grace Bio-Lab, Bend, OR, USA) containing a well at its center was gently placed on a coverslip. A volume of 7 µL of one of the previously prepared solutions was deposited in the well, and a microscope slide was slowly placed over it, without pressure. This assembly produced a thin layer of emulsion for observation. The excitation and emission wavelengths for each of the fluorophores were chosen according to Gallier et al. [47].

### 2.6. Statistical Analysis

A statistics analysis of the results was performed using Statistical Analysis System (SAS) software (SAS University edition, version 3.5, Cary, NC, USA) on three replicates for each experiment. The composition and quantification of phosvitin in the fractions were analyzed by one-way analysis of variance (ANOVA) and the Tukey test for multiple comparisons. The particle size distribution was analyzed by multifactorial ANOVA using the MIXED procedure. The creaming index was analyzed by a *t*-test. A confidence interval of 95% (*p* < 0.05) was used for all tests. Data were expressed as mean ± standard deviation (SD).

## 3. Results and Discussion

### 3.1. Ultrafiltration Performance

**Figure 2:**
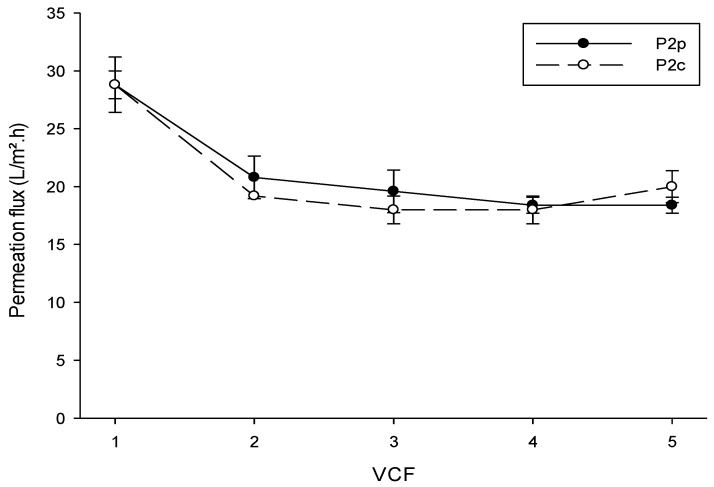
Permeation flux obtained during UF of control and pressure-treated egg yolk plasmas at different volume concentration factors (VCFs). P2_C_: control plasma, P2_P_: plasma from pressure-treated granules.

Permeation flux as a function of VCF for control plasma (P2_C_) and plasmas from pressure-treated granules (P2_P_) is presented in Figure 2. The permeation fluxes of both plasmas were not significantly different at each VCF (*p* > 0.05). At 1×, the permeation flux was 29 L/m^2^·h, which is lower than values results presented by Chay Pak Ting et al. [45] (50 L/m^2^·h) during UF (10 or 30 kDa) of crude phosphvitin solutions (5.0% *w*/*v*), with another cross-flow UF system. As the feed was concentrated, the permeation flux decreased to 20 L/m^2^·h at 2×, but remained stable until the end of UF (VCF = 5×). Adversely, the permeation fluxes observed by Chay Pak Ting et al. [45] decreased by 48% at a VCF of 6×. In the latter study, however, UF was conducted at 50 °C, which possibly increased membrane fouling incidence due to a lower calcium phosphate solubility at this temperature. In this study performed at room temperature, the presence of phosvitin in P2_P_ did not seem to affect UF performance. Phosvitin is negatively charged, as is the PES membrane [45], therefore, the electrostatic repulsions between phosvitin and the membrane material prevent fouling at the membrane surface, allowing the flux to remain constant.

### 3.2. Impact of HHP and UF on Fraction Compositions

#### 3.2.1. Protein and Lipid Composition

The protein and lipid concentrations of all fractions are presented in Table 1 and are expressed on a dry basis. Fractions G1 (initial granule), G2_C_ (control granule) and G2_P_ (pressure-treated granule) have the same protein and fat contents (*p* > 0.05). The G1 fraction had 63.4 ± 0.32% *w*/*w* and 27.9 ± 0.8% *w*/*w* of protein and lipid, respectively, which agrees with the literature [42,46]. Plasmas P2_C_ and P2_P_ also had the same protein content (*p* > 0.05), but P2_C_ had a higher lipid content than P2_P_ (22.7 ± 1.4% *w*/*w* vs. 16.1 ± 0.4% *w*/*w*) (*p* > 0.05). The 10 kDa membrane retained almost all the lipids, with R_P_ containing more lipids than R_C_ (*p* > 0.05). The resulting permeates, Pm_C_ and Pm_P_, contained only traces of lipid at 0.23 ± 0.11% *w*/*w* and 0.05 ± 0.05% *w*/*w*, respectively. Similarly, most proteins were retained by the 10 kDa membrane, and the protein contents of R_C_ and R_P_ were higher than their corresponding plasmas (*p* < 0.05). UF-Permeates Pm_C_ and Pm_P_ had similar protein contents of 40.7 ± 0.8% *w*/*w* and 37.1 ± 0.7% *w*/*w*, respectively (*p* > 0.05). Dumas’ method measures the total nitrogen content of a sample; therefore, these values might correspond to peptides rather than proteins. Globally, only few compositional differences were observed between control and pressure-treated fractions, suggesting HHP does not impact the total protein and total lipid contents.

#### 3.2.2. Protein Profiles

**Figure 3:**
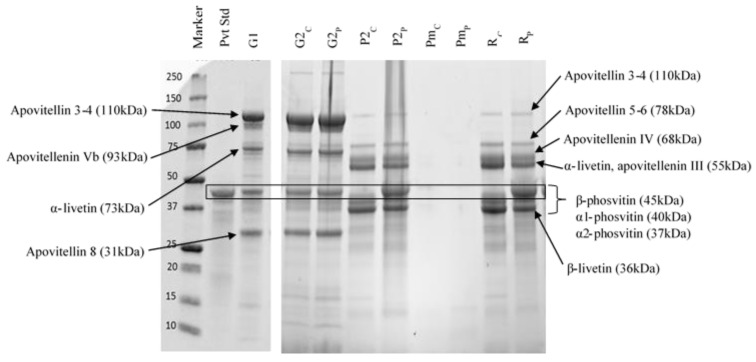
Protein profiles of control and pressure-treated fractions of egg yolk obtained by sodium dodecyl sulfate polyacrylamide gel electrophoresis (SDS-PAGE). Pvt Std: commercially phosvitin standard, G1: initial granule, G2_C_: control granule, P2_C_: control plasma, G2_P_: pressure-treated granule, P2_P_: plasma from pressure-treated granules, Pm_C_: control UF-permeate, R_C_: control UF-retentate, Pm_P_: UF-permeate of plasma from pressure-treated granules, R_P_: UF-retentate of plasma from pressure-treated granules.

Protein profiles obtained by SDS-PAGE for all fractions and phosvitin standard are presented in Figure 3. The protein bands were identified according to their molecular weights from previous studies [10,37,43] The phosvitin standard was composed of three major bands corresponding to its three isoforms, β-phosvitin (45 kDa), α1-phosvitin (40 kDa) and α2-phosvitin (37 kDa). As the most abundant isoform, β-phosvitin had a much higher intensity band than α1- or α2-phosvitin. The three granules G1, G2_C_ and G2_P_ had the same protein profiles, with 4 major bands identified as apovitellins 3–4 (110 kDa), α-livetin (73 kDa), β-phosvitin (45 kDa) and apovitellin 8 (31 kDa). Both control and pressure-treated G2 contained β-phosvitin, suggesting that the pressurization treatment does not transfer all the phosvitin to the plasma and some remains trapped in the granular network. Indeed, a more severe HHP treatment (600 MPa for 10 min) has been shown to produce better phosvitin recovery [43]. The protein profiles of Pm_C_ and Pm_P_ did not have visible bands. Theoretically, they should mostly contain molecules of less than 10 kDa, such as peptides. The retentates had the same profiles as their corresponding plasmas, except that the intensity of the bands was higher since UF concentrated the plasmas. For these fractions, seven major bands were detected and identified as apovitellin 3–4 (110 kDa), apovitellin 5–6 (78 kDa), apovitellenin IV (68 kDa), apovitellenin III (55 kDa), α-livetin (55 kDa), α1-phosvitin (40 kDa), α2-phosvitin (37 kDa) and β-livetin (36 kDa). A high intensity band corresponding to β-phosvitin (45 kDa) was present in plasma and retentate pressure-treated samples, but absent in plasma and retentate controls. Therefore, the application of an HHP treatment of 400 MPa for 5 min on G1 specifically induced the transfer of β-phosvitin.

According to these observations, α1 and α2-phosvitin were transferred to the plasma by the simple centrifugation of G1 but β-phosvitin was not. In contrast, the combination of centrifugation and HHP released the β-phosvitin from the granular network. Duffuler [43] observed the same phenomenon with all HHP parameters tested (400 and 600 MPa for 5 and 10 min). Phosvitin is bound to HDL by phosphocalcic bridges to form the compact structure of the granule. However, isoforms α1 and α2 contain only 3% of phosphorus, compared to 10% in β-phosvitin [2]. Consequently, the bond between HDL and α-phosvitin is weaker and can be destabilized by centrifugal force. For β-phosvitin, a greater force, such as pressure, is required to disrupt the phosphocalcic bridges with HDL. In addition, since phostivin is water-soluble, due to its serine residues, it can easily move into the plasma once released from the granular structure [37,38,43].

#### 3.2.3. Mineral Composition

The phosphorus and iron contents of all fractions are presented in Table 2 and expressed on a dry basis. Since phosvitin is a phosphorylated protein, a high concentration of phosphorus may be an indication of the presence of phosvitin in a fraction [5]. Similarly, most of the iron in egg yolk is bound to phosvitin; therefore, a high concentration of iron could also indicate the presence of phosvitin [8].

The G1 fraction seemed to contain twice the phosphorus content of G2_C_ and G2_P_ (0.65 ± 0.24% *w*/*w* vs. 0.33 ± 0.09% *w*/*w* and 0.30 ± 0.04% *w*/*w*, respectively). However, no significant difference was observed in their phosphorus and iron contents (*p* > 0.05), which agrees with Duffuler [43]. Nevertheless, egg yolk granule contains phospholipids such as phosphatidylcholine and phosphatidylethanolamine, which could induce high phosphorus content in the granule fractions, despite the reduction of the phosvitin content [42]. The similar mineral content of G2_C_ and G2_P_ may be because HHP did not induce complete transfer of phosvitin to plasma, as shown in the SDS-PAGE profiles.

Except for the granule samples, all fractions from pressure-treated granules (P2_P_, R_P_ and Pm_P_) had higher phosphorus contents than their corresponding control fractions (P2_C_, R_C_ and Pm_C_), suggesting the presence of phosvitin in these fractions. The Pm_P_ fraction had a high phosphorus content, but FPLC analysis showed that no phosvitin was detected in this fraction. This high phosphorus content corresponded to the presence of phosvettes, small peptides (≤16 kDa) generated from phosvitin [48]. The P2_P_ and R_P_ also had higher iron contents than controls P2_C_ and R_C_. However, Pm_P_ had a lower iron content than P2_P_ and R_P_. During UF, the iron remained in the retentate, since it is mostly bound to phosvitin. Therefore, HHP treatment (400 MPa for 5 min) did not affect the phosvitin-iron bond, demonstrating the great stability of phosvitin under pressure, as revealed by Castellani et al. [44].

#### 3.2.4. Phosvitin Analysis

##### Fast Protein Liquid Chromatography

**Figure 4:**
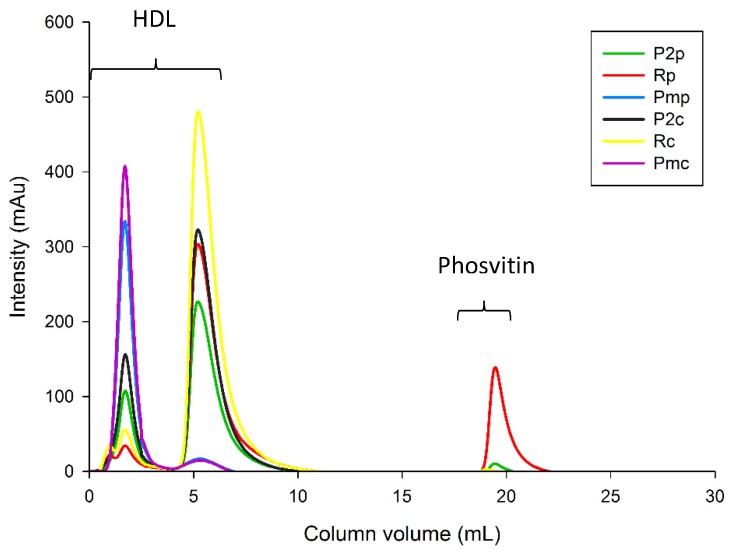
Chromatograms obtained by fast protein liquid chromatography (FPLC) for control and pressure-treated plasma, retentate and permeate fractions. P2_C_: control plasma, P2_P_: plasma from pressure-treated granules, Pm_C_: control UF-permeate, Pmp: UF-permeate of plasma from pressure-treated granules, R_C_: control UF-retentate, R_P_: UF-retentate of plasma from pressure-treated granules.

The phosvitin contents of all fractions are expressed on a dry basis and presented in Table 3. A phosvitin peak was identified at 19 mL of CV, with the use of phosvitin standard solution (Figure 4). Two major peaks were observed at 2.0 and 5.5 mL of CV and were identified as HDLs [32,43].

Phosvitin was not detected as β-phosvitin in the protein profiles of P2_C_, Pm_C_ and Pm_P_. However, a very low concentration of phosvitin was detected by FPLC in R_C_ (0.12 ± 0.31% *w*/*w*), while β-phosvitin was not visible in its protein profile. The P2_C_ fraction could have contained a trace amount of phosvitin and UF increased its concentration in the retentate. The G2_P_ fraction had significantly less phosvitin than G1, but not G2_C_, as seen in Table 3 (*p* > 0.05). This result suggests that phosvitin was probably lost during the centrifugation process to prepare G2 and P2 fractions. The G2_P_ fraction had less phosvitin than the G2_C_ fraction, indicating that HHP induced the transfer of some phosvitin (*p* > 0.05). However, only about a third is transferred, since the G2_P_ fraction still had 6.58 ± 0.21% *w*/*w* phosvitin. The R_P_ fraction had a higher phosvitin content than P2_P_ (26.00 ± 4.12% *w*/*w* vs. 1.73 ± 0.07% *w*/*w*) (*p* > 0.05), confirming the retention of phosvitin by the UF membrane, and its concentration in the retentate, as observed by Chay Pak Ting et al. [45] Therefore, the combination of HHP and UF is an interesting technique to obtain a phosvitin-enriched fraction. However, the amount of phosvitin in P2_P_ was lower than the 24.14 ± 5.21% *w*/*w* (dry basis), obtained by Duffuler in a plasma fraction using the same pressurization treatment (400 MPa for 5 min) [43]. This was presumably due to the different dilution ratios, 1:10 in this study vs. 1:100 in Duffuler, used to solubilize the granule fraction [43]. Consequently, the choice of the ratio for granule solubilization may be a determining parameter for the recovery of phosvitin following pressurization. A larger ratio might better hydrate the granule, which might also allow increased water entry into the granular network during HHP treatment. This would result in the disruption of more phosphocalcic bridges of the complex HDL-phosvitin of the granule and increased phosvitin release from the network to the plasma [38], thus enabling its purification by UF.

Chromatograms of plasma, retentate and permeate fractions are presented in Figure 4. In addition to the phosvitin peak, two peaks were identified at 2.0 and 5.5 mL of CV in plasma fractions, which correspond to HDL according to Ren and Wu [32]. The peaks identified as HDL were separated after UF treatment. The peak at CV = 2.0 mL was recovered in UF-permeates, while the peak at CV = 5.5 mL was recovered in retentates. Therefore, the 10 kDa PES membrane induced the selective separation of these HDL molecules.

##### LC-MS/MS Analysis

The peaks obtained by fast protein liquid chromatography (FPLC) analysis were identified through proteomic analysis of the P2_P_, R_P_ and Pm_P_ fractions (Table 4). Vitellogenin-1 (Vit-1) and vitellogenin-2 (Vit-2) are precursors of various egg yolk proteins, especially HDL and phosvitin. The proportion of Vit-1 was higher in the P2_P_ and R_P_ fractions than in Pm_P_, where no Vit-2 was detected_._ This confirmed the presence of phosvitin in the P2_P_ fraction and its retention by the 10 kDa UF membrane in the R_P_ fraction. As mentioned previously, the detection of Vit-1 in the Pm_P_ fraction could be due to the presence of phosvettes [48]. In addition, this analysis provides additional information to identify the peaks at 2.0 and 5.5 mL of CV of the chromatogram (Figure 4). According to the total spectrum count, the peak recovered in the Pm_P_ fraction at 2.0 mL of CV might be apolipoprotein A-I and the peak recovered in R2_P_ fraction at 5.5 mL of CV may consist of apolipoprotein B, ovotransferrin and serum albumin. Apolipoproteins A-I and B are constituents of HDL [49], in accordance with the studies of Ren and Wu [32] and Duffuler [43]. In contrast, ovotransferrin is an egg white protein [50] and serum albumin is recovered in plasma egg yolk [42], suggesting the presence of these protein contaminants during egg separation and the first egg yolk centrifugation.

### 3.3. Emulsifying Properties

#### 3.3.1. Particle Size Distribution

**Figure 5:**
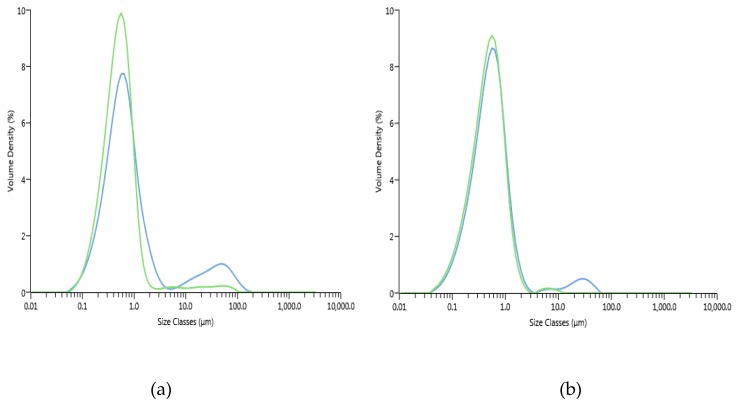
Distribution size of oil droplets of emulsions from control UF-retentates (**a**) and UF-retentates of plasma from pressure-treated granules (**b**) emulsions at 0 h after preparation, without tris/glycine/SDS (blue line) and with SDS added (green line).

**Figure 6:**
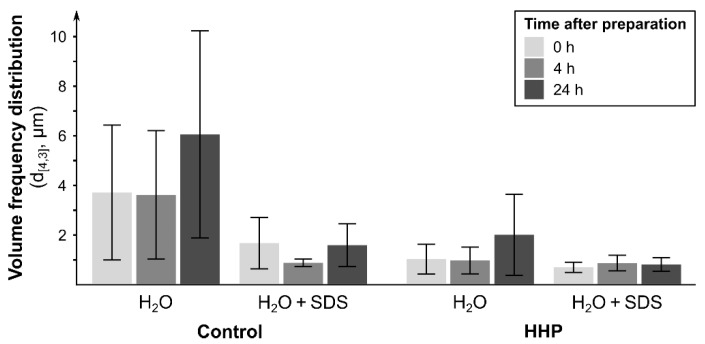
The volume frequency distribution (d_[4,3]_) of emulsions from control UF-retentate and UF-retentate of plasma from pressure-treated granules 0 h, 4 h and 24 h after emulsion preparation (Tukey’s test, α = 0.05, *n* = 3, ±SD).

The size distribution of oil droplets and their volume frequency distribution (d_[4,3]_) are presented in Figure 5 and Figure 6, respectively.

The size distribution of oil droplets showed that for both control and pressure-treated emulsions, there was a major population around 0.6 µm immediately after preparation. The control emulsion had a second population around 80 µm, affected by the addition of SDS, suggesting the presence of flocs. Emulsions made with pressure-treated retentate also had a second population of oil droplets following the addition of SDS, but these were smaller, which indicates an emulsion more stable to flocculation.

Despite differences in size distributions, the addition of SDS did not have a statistically significant effect on the d_[4,3]_, for both emulsion from control and emulsion from pressure-treated ingredient (*p* > 0.05). For example, at 0 h, the control decreased from 3.69 ± 2.72 µm to 1.65 ± 1.04 µm following the addition of SDS. Comparatively, the emulsion from pressure-treated ingredient experienced a smaller decrease from 1.00 ± 0.60 µm to 0.67 ± 0.21 µm. Following the addition of SDS, the larger the decrease in d_[4,3]_, the less stable the emulsion. A smaller decrease, such as that obtained with the emulsion made with UF-retentate of plasma from pressure-treated granules, produces an emulsion less prone to flocculation.

#### 3.3.2. Creaming Index

The creaming index indicates the stability of an emulsion against creaming. The higher the creaming index, the more sensitive the emulsion is to creaming and, therefore, unstable. The creaming index of the control emulsion reached 93.75% and that of the one containing UF-retentate of plasma from pressure-treated granules was only 6.25%. The control emulsion was clearly more prone to creaming than the pressure-treated emulsion (*p* < 0.05). Previously, the measurement of d_[4,3]_ demonstrated that the control emulsion flocculates more rapidly than the emulsion containing UF-retentate of plasma from pressure-treated granules. As mentioned, the size distribution of the oil droplets is largely the same for both emulsions, with a major population around 1 µm (Figure 5). Thus, the size of the oil droplets is not the main parameter contributing to the greater stability of the emulsion made with UF-retentate of plasma from pressure-treated granules.

#### 3.3.3. Protein Profiles of Emulsion Fractions

**Figure 7:**
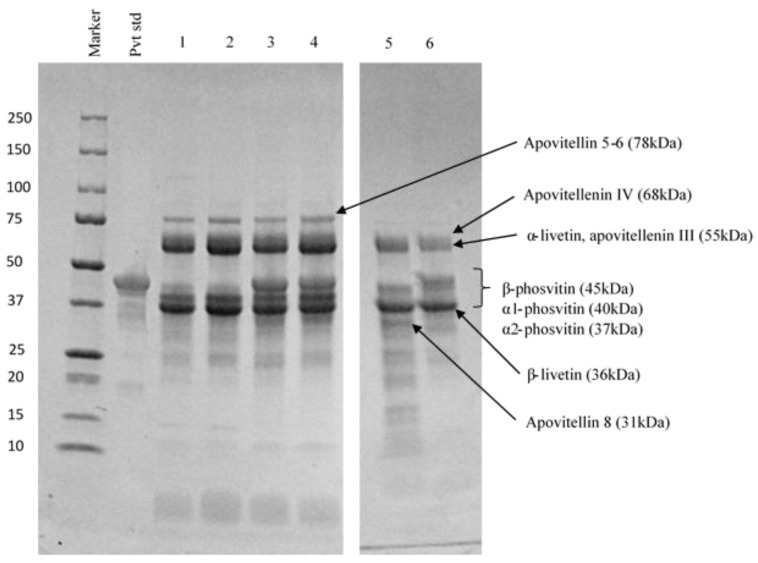
Protein profiles by SDS-PAGE of the cream and aqueous phases of emulsions from control UF_retentate and UF-retentate of plasma from pressure treated granules. Pvt Std: commercial phosvitin standard, 1: control emulsion, 2: control aqueous phase, 3: emulsion containing UF-retentate of plasma from pressure-treated granules, 4: aqueous phase of emulsion containing UF-retentate of plasma from pressure-treated granules, 5: control cream, 6: cream from emulsions containing UF-retentate of plasma from pressure-treated granules.

The protein profiles of the entire emulsion, cream and aqueous phases of the control emulsion and of the emulsion containing UF-retentate of plasma from pressure-treated granules are presented in Figure 7. The protein profiles of creams show which proteins are adsorbed at the interface of oil droplets. As expected, no β-phosvitin was found in the emulsion, cream or aqueous phase of the control. In contrast, β-phosvitin was observed in the cream and aqueous phases of the emulsions containing UF-retentate of plasma from pressure-treated granules, suggesting that only some of the β-phosvitin is adsorbed at the interface. In addition, the protein profile of cream from the emulsion containing UF-retentate of plasma from pressure-treated granules shows various bands (apovitellenin IV (68 kDa), apovitellenin III (55 kDa), α-livetin (55 kDa), β-phosvitin (45 kDa), β-livetin (36 kDa)), implying that a group of proteins stabilizes the emulsion. However, as the creaming index indicated, the presence of β-phosvitin had a large impact on the stability of that emulsion to creaming compared to the non-pressurized plasma sample.

#### 3.3.4. Confocal Microscopy

**Figure 8:**
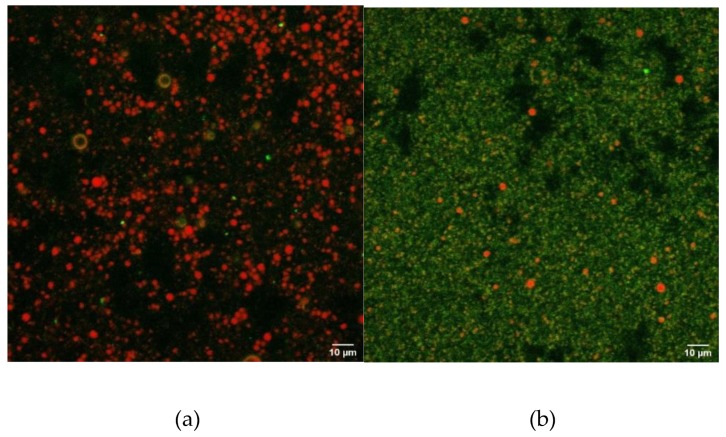
Images obtained by confocal laser scanning microscopy of emulsions containing control UF-retentate (**a**) and UF-retentate of plasma from pressure-treated granules (**b**) and targeted by Fast Green and Nile Red to observe proteins and fat, respectively.

Images obtained by confocal laser scanning microscopy (CLSM) are presented in Figure 8. The proteins (green) were labelled with Fast Green, while the lipids (red) were labelled with Nile Red. Prior to CLSM observations, the oil droplets were washed using a sucrose layering technique to avoid interference with the dispersed protein in the aqueous phase. In the washed cream obtained from an emulsion containing UF-retentate of plasma from pressure-treated granules, small oil droplets were trapped in a compact protein network. In contrast, the cream obtained from the control emulsion contained oil droplets of heterogeneous size that tended to form flocs, while less protein was observed. HHP caused the denaturation and aggregation of proteins and this network stabilized the emulsion, since the oil droplets could not interact and flocculate. The cream should have had a low protein content because of the sucrose wash step, which used density differences to isolate oil droplets and proteins at the interface during the preparation of slides for microscopy (Section 2.5.5). Instead, on centrifugation, the aggregated proteins formed a gel network, trapping oil droplets that were recovered at the top of the sucrose layer. Indeed, the cream layer recovered from the emulsion made with UF-retentate of plasma from pressure-treated granules seemed more compact than that obtained from the control emulsion, suggesting the formation of a protein gel. Consequently, according to these images, it is difficult to attribute the greater stability of the emulsion made with UF-retentate of plasma from pressure-treated granules only to phosvitin. The effect of pressure-induced aggregation of the plasma proteins appears to have a role in stabilizing the emulsion. Several studies demonstrated that an HHP treatment can increase the stability of an emulsion due to the modification of proteins [51,52,53]. Additionally, emulsion gelation is a known technique to encapsulate oil droplets, increasing stability [54]. HHP treatment (400 MPa for 5 min) may have denatured the egg yolk granule proteins and caused the formation of a gel due to hydrophobic interactions inducing the encapsulation of the oil droplets in the gel.

## 4. Conclusions

These results suggest that the combination of HHP and UF might be interesting for industrial purposes for the production of a fraction enriched in phosvitin. The combined techniques allow the selective recovery and concentration of phosvitin in the retentate, as seen with FPLC and proteomic analysis. Indeed, industrialization of HHP process (batch process) remains a challenge, but further work will determine how this purification method could be scaled up, and how profitable it could be.

Emulsions made with UF-retentate of plasma from pressure-treated granules are more stable to flocculation and creaming than non-pressurized emulsions. However, CLSM and protein profiles of emulsions demonstrated that oil droplets were trapped in a gel-like network rather than stabilized specifically by phosvitin. Additional analyses are needed to provide further evidence of the molecule(s) responsible for the emulsifying properties of the UF-retentates of plasma from pressure-treated granules.

## Figures and Tables

**Table 1:** Protein and lipid contents of control and pressure-treated fractions of egg yolk.

Samples		Protein	Lipid
		(% *w*/*w*, Dry Basis)	
Initial granule	G1	63.4 ± 0.3 ^a, b^	27.9 ± 0.8 ^c^
Control	G2_C_	64 ± 1.1 ^a^	26.8 ± 0.4 ^c^
	P2_C_	54.1 ± 0.5 ^c^	22.7 ± 1.4 ^d^
	Pm_C_	40.7 ± 0.8 ^d^	0.23 ± 0.11 ^f^
	R_C_	62.9 ± 0.5 ^a, b^	36.8 ± 1.07 ^b^
Pressure-treated	G2_P_	64.5 ± 0.4 ^a^	28.2 ± 0.2 ^c^
	P2_P_	55.6 ± 1.2 ^b, c^	16.1 ± 0.4 ^e^
	Pm_P_	37.1 ± 0.7 ^d^	0.05 ± 0.05 ^f^
	R_P_	52.9 ± 0.9 ^b, c^	41.5 ± 3.4 ^a^

Data with different letters (a–f) within each column are significantly different (Tukey test, α = 0.05, *n* = 3). G1: initial granule, G2_C_: control granule, P2_C_: control plasma, G2_P_: pressure-treated granule, P2_P_: plasma from pressure-treated granule, Pm_C_: control UF-permeate, R_C_: control UF-retentate, Pm_P_: UF-permeate of plasma from pressure-treated granules, R_P_: UF-retentate of plasma from pressure-treated granules.

**Table 2:** Phosphorus and iron contents of control and pressure-treated fractions of egg yolk.

Samples		Phosphorus (P)	Iron (Fe)
		% *w*/*w*	×10^−2^% *w*/*w*
Initial granule	G1	0.65 ± 0.24 ^d, e^	0.50 ± 0.12 ^c^
Control	G2_C_	0.33 ± 0.09 ^e^	1.20 ± 0.33 ^c^
	P2_C_	0.98 ± 0.10 ^c, d^	0.49 ± 0.18 ^c^
	Pm_C_	1.52 ± 0.09 ^b, c^	1.31 ± 1.19 ^c^
	R_C_	0.50 ± 0.10 ^d, e^	0.42 ± 0.19 ^c^
Pressure-treated	G2_P_	0.30 ± 0.04 ^e^	1.20 ± 0.16 ^c^
	P2_P_	2.74 ± 0.11 ^a^	6.31 ± 0.70 ^a^
	Pm_P_	2.97 ± 0.27 ^a^	0.50 ± 0.10 ^c^
	R_P_	2.07 ± 0.53 ^b^	3.20 ± 0.98 ^b^

Data with different letters (a–e) within each column are significantly different (Tukey test, α = 0.05, *n* = 3). G1: initial granule, G2_C_: control granule, P2_C_: control plasma, G2_P_: pressure-treated granule, P2_P_: plasma from pressure-treated granules, Pm_C_: control UF-permeate, R_C_: control UF-retentate, Pm_P_: UF-permeate of plasma from pressure-treated granules, R_P_: UF-retentate of plasma from pressure-treated granules.

**Table 3:** Phosvitin content of control and pressure-treated fractions of egg yolk.

Samples		Phosvitin
		% *w*/*w*, Dry Basis
Initial granule	G1	12.40 ± 0.34 ^b^
Control	G2_C_	9.25 ± 0.82 ^b, c^
	P2_C_	ND
	Pm_C_	ND
	R_C_	0.12 ± 0.31 ^d^
Pressure-treated	G2_P_	6.58 ± 0.21 ^c^
	P2_P_	1.73 ± 0.07 ^d^
	Pm_P_	ND
	R_P_	26.00 ± 4.12 ^a^

Data with different letters (a–d) within each column are significantly different (Tukey test, α = 0.05, *n* = 3). G1: initial granule, G2_C_: control granule, P2_C_: control plasma, G2_P_: pressure-treated granule, P2_P_: plasma from pressure-treated granules, Pm_C_: control UF-permeate, R_C_: control UF-retentate, Pm_P_: UF-permeate of plasma from pressure-treated granules, R_P_: UF-retentate of plasma from pressure-treated granules.

**Table 4:** Proteomic analysis of fractions generated from pressure-treated granules: plasma P2_P_, UF-retentate R_P_ and UF-permeate Pm_P_.

Accession Number	Identified Proteins	Molecular Weight (kDa)	Sequence Coverage (%)	Total Spectrum Count		
				P2_P_	R_P_	Pm_P_
VIT2_CHICK	Vitellogenin-2, OS: *Gallus gallus*, OX = 9031, GN = VTG2, PE = 1, SV = 1	205	58	1017	1048	0
A0A1D5NW68_CHICK	Serum albumin, OS = *Gallus gallus*, OX = 9031, GN = ALB, PE = 3, SV = 1	70	84	759	786	132
A0A1D5NUW2_CHICK	Vitellogenin-1, OS = *Gallus gallus*, OX = 9031, GN= VTG1, PE = 4, SV = 1	211	53	627	584	286
A0A1D5P4L7_CHICK	Ovotransferrin, OS = *Gallus gallus*, OX =9 031, GN = TF, PE = 3, SV = 1	78	84	329	336	36
F1NV02_CHICK	Apolipoprotein B, OS = *Gallus gallus*, OX = 9031, GN = APOB, P E= 4, SV = 2	523	38	212	166	91
A0A1L1RJF5_CHICK	Apolipoprotein A-I, OS = *Gallus gallus*, OX = 9031, GN = APOA1, PE = 3, SV = 1	32	55	37	27	157

P2_P_: plasma from pressure-treated granules, Pm_P_: UF-permeate or plasma from pressure-treated granules, R_P_: UF-retentate of plasma from pressure-treated granules. OS: organism name, OX: organism identifier, GN: gene name, PE: protein existence, SV: sequence version, ALB: albumin, TF: transferrin, VTG1: vittelogenin-1, VTG2: vittelogenin-2, APOB: apolipoprotein B, APOA1: apolipoprotein A-I.
